# Sparsely distributed contours dominate extra-striate responses to complex scenes

**DOI:** 10.1016/j.neuroimage.2008.04.266

**Published:** 2008-08-15

**Authors:** Serge O. Dumoulin, Steven C. Dakin, Robert F. Hess

**Affiliations:** a McGill Vision Research Unit, Department of Ophthalmology, McGill University, Montréal, Canada; b Institute of Ophthalmology, University College London, London, UK

**Keywords:** Brain imaging, fMRI, Natural image, Texture, Vision, Visual cortex

## Abstract

The human visual system exploits redundancy in natural scenes to derive useful information. Such redundancy is frequently associated with either contours or textures within images. In this study we use fMRI to evaluate how the total amount of contrast-energy contained in contours and textures within natural images affect responses in visual cortex. We used both the entire natural image and parts of it containing predominantly contour or texture information. We modified these natural images in order to match other image properties that are known to affect cortical responses as closely as possible. These modified natural images, i.e. pseudo-natural images, remain highly recognizable. We also used synthetic images without recognizable content but with closely matched image properties. We report that most of the primary visual cortex (V1) signal variations are explained by the total amount of contrast-energy in the images. Extra-striate visual cortex, on the other hand, is driven strongest by images containing sparsely distributed contours; independent of contrast-energy amount or recognizable image content. These results provide evidence for an initial representation of natural images in V1 based on local oriented filters. Later visual cortex (and to a modest degree V1) incorporates a facilitation of contour-based structure and suppressive interactions that effectively amplify sparse-contour information within natural images.

## Introduction

**Fig. 1:**
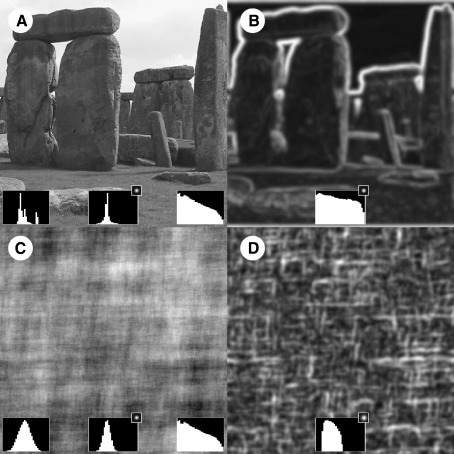
(A) A natural scene with (left inset) a histogram of its luminances, (middle inset) a histogram of the responses of a derivative filter (Laplacian-of-Gaussian, shown at top right of inset), and (right inset) a log–log plot of the power spectrum of the image. (C) As (A) but with a “phase-scrambled” version of the natural image. Note that, although the power spectra of (A) and (C) are identical, the histograms of luminance and filter responses from the original image are more “peaky” than for the phase-scrambled version. In other words, the distributions are more *sparse*. The cause of the discrepancy in filter responses is the distribution of local contrast-energy in the two patterns as depicted in (B,D). This shows the spatial distribution of local RMS contrast-energy within (A) and (C) computed using the Gaussian weighting kernel depicted in the top right of the inset. The histogram of local RMS energy (inset) for the natural image is quite flat indicating a relatively uniform distribution of filter responses ranging from very weakly to very strongly. In contrast, the phase-scrambled counterpart has a narrower range of filter responses. Judging by the contrast-image shown in (B) the difference in luminance and filter-response histograms between natural and phase-scrambled images appear to be due to the presence of extended contour structure in (A).

Understanding vision centers on understanding how we extract useful information from an extraordinarily rich source, like a natural image (Fig. 1A), to guide behavior. What properties of natural images are relevant (for a review of this area see Simoncelli and Olshausen, 2001; Srivastava et al., 2003)? Neurons in primary visual cortex (V1 or striate cortex) respond to a relatively narrow range of orientations within small (local) regions of the visual field (Hubel and Wiesel, 1968) (although they are influenced by neighboring neurons Allman et al., 1985; Fitzpatrick, 2000). As such, V1 can be thought of as representing the outside world like a bank of oriented filters (De Valois and De Valois, 1990). Given that the structure of the human visual system is probably optimized to process the structure of the visual world, we must consider the local structure of natural images because early visual processing is local.

Consider a natural image (Fig. 1A) and a version of it where the Fourier phase-spectrum has been randomized (C). These images have identical *global* power spectra (right inset of panels A and C) but an analysis of their local contrast structure, reveals clear differences (middle inset of panels A and C; local contrast is computed using a Laplacian-of-Gaussian filter, shown in the top right). The local contrast-histogram is more “peaky” in the natural image compared its phase-scrambled counterpart. A more “peaky” distribution indicates that few local analyzers respond strongly and many more respond weakly, i.e. the distribution is more *sparse*. While the distribution of local contrast-energy in phase-scrambled image (D) is relatively uniform, in natural images (B) local contrast-energy is concentrated around the edges (or contours) and is punctuated by regions of relatively low energy within surfaces. This distribution of local contrast-energy around the edges leads to the sparse filter-response histogram in natural images. Sparseness arising from contour structure is a key but poorly understood physical property of natural images.

This leads us to our research question: how does image sparseness affect visual processing of natural images and how does this relate to the presence of contours (which we propose is related to sparseness in natural images, see previous paragraph)? We use an fMRI paradigm to explore this question. Previous fMRI studies examining the effect of natural image statistics on brain activation have used natural images and phase-scrambled counterparts (Dakin et al., 2002; Olman et al., 2004; Rainer et al., 2001). As described above, phase scrambling preserves global properties of natural images but drastically changes their local properties, transforming sparse/locally-high contrast images (Figs. 1A,B) into dense/locally-low contrast *textures* (Figs. 1 C,D), i.e. images where the individual positions of features is unimportant. Thus, phase scrambling turns images, that contains a mixture of dense-texture and sparse-contours, into dense textures. Consequently, one cannot distinguish whether the addition of texture or the subtraction of contour structure underlies phase-scrambling manipulations.

To resolve this issue a second aim of the current investigation was to explicitly examine the relative importance of texture, contour, contrast-energy and feature sparseness in early visual cortex. We used both the entire natural image and parts of it containing predominantly contour or texture information. We modified these natural images in order to match other image properties that are known to affect cortical responses as closely as possible (such as contrast-energy). These modified natural images, i.e. pseudo-natural images, remain highly recognizable. To preempt our findings, we report a large difference in response to these images in striate (V1) and extra-striate visual cortex. The response of V1 is related to the total contrast-energy in the images, whereas extra-striate visual cortex is driven strongest by images containing sparsely distributed contours, independent of the amount of contrast-energy or recognizable image content. We propose that these findings are consistent with an initial representation of natural images in V1 based on local oriented filters, with later visual cortex (and to a modest degree V1) incorporating facilitation and suppressive interactions to effectively amplify response to sparse-contour information.

## Materials and methods

### Subjects

Six experienced psychophysical observers were used as subjects (2 female, mean age: 38, age range: 27–54), four of whom were naive to the purpose of the study. The subjects were instructed to fixate at a provided fixation point and trained prior to the scanning session to familiarize them with the task. All observers had normal or corrected-to-normal visual acuity.

### Visual stimuli

The visual stimuli were generated in the MatLab programming environment using the PsychToobox (Brainard, 1997; Pelli, 1997) on a Macintosh G4 Powerbook, and displayed on a LCD projector (NEC Multisync MT820). The total visual display subtended 20 degrees.

**Fig. 2:**
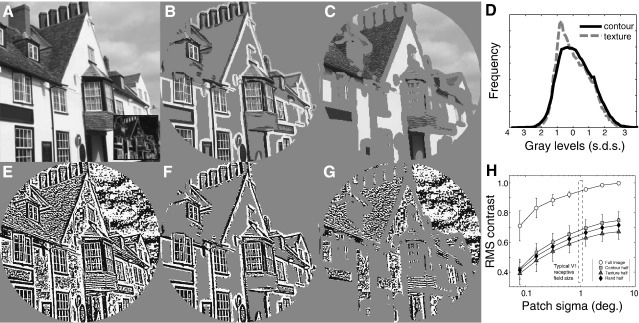
Examples of natural visual stimuli used. (A) A gray scale image with an orientation energy map indicating pixels with maximal local orientation energy (inset). Pixels in image (A) were placed in either the “contour” image (B) or “texture” image (C), based upon the local orientation energy of image (A, inset). (D) Histograms of gray levels extracted from regions of the original image (averaged over 200 images) associated with contours (black line) and texture (gray dotted line) respectively. (E) Binary image created from image (A). (F,G) “Contour” and “texture” images extracted from image (E). In the fMRI experiments only images (E–G) were used. (H) From a statistical perspective, the binary images have many advantages over unmodified natural scenes, the most important of which is likely their local contrast statistics. Applying a local contrast analysis (similar to Figs. 1B,D) across many local region sizes, one can see that half-images has similar local contrast statistics to one another (compared to the differences between full- and half-images) across all scales of analysis. This is a key requirement, given the likely importance of such local statistics, if one is to isolate the role of contour structure in driving cortical response.

The stimuli were derived from natural images (*n* = 200, see for example Fig. 2A). Natural images vary in a wide range of image statistics, such as RMS contrast and sparseness, which are known to affects the fMRI response at various stages within the human visual pathway (e.g. Boynton et al., 1999; Olman et al., 2004; Sclar et al., 1990). In examining the differential fMRI response to contour-defined and texture-defined structure within natural scenes it was therefore imperative that our stimuli were sufficiently well matched to rule out any explanation of our findings based on systematic variation in such image statistics. To this end, we examined whether RMS contrast and/or kurtosis might vary with the presence of contour or texture structure within our natural scene set. We did this by filtering 200 natural images (e.g. Fig. 2A), by setting half the image pixels to mean luminance according to the energy of a bank of local oriented filters (Fig. 2A inset). This allowed us to separately collate all those image pixels that fell within contoured (B) and textured (C) regions, and construct histograms of their gray levels (D). Results revealed that for images normalized to have unit RMS contrast the RMS contrast of contour-consistent image structure was on average around 8% higher than for image structure falling outside of the contoured regions (RMS values of 1.034 and 0.96 respectively). We also found that the kurtosis of image consistent structure was on average 5% higher than other more textured regions of the scene (+ 2.75 and + 2.62 respectively). These differences convinced us of the need to match the statistics of the contoured and textured regions.

We matched the statistical properties of contoured and texture regions by binarizing the images. We refer to these binarized images as pseudo-natural images to reflect that the images are derived from natural images and remain highly recognizable. Binarization was achieved using a lowpass-filtered version of the image (*σ* = 2 pixels). The pixels of the binarized image were set to maximum or minimum luminance depending on whether a pixel in the original image was greater or less than the corresponding pixel in the low-pass image. This maximizes luminances greater than the local mean and minimizes luminances below the local mean. The low-pass image was scaled to ensure that this comparison led to half of the pixels in the binarized image being black and half white (Fig. 2E). We chose this procedure for the following reasons. First, it maximizes Michelson and RMS contrast for a given image, ensuring that our stimuli would elicit robust fMRI signals. Second, contour, texture, and indeed the whole scene remain highly recognizable suggesting that key image structure has been retained. Third, binarization closely matches RMS and kurtosis statistics. Fourth, using a similar procedure to divide the original broadband images (e.g. Figs. 2F and G) using local-energy produces robust contours but non-contoured regions are frequently dominated by uniform regions whose boundaries generate “false edges” themselves (i.e. region edges in panel C). Binarization of band-pass filtered images tends to force pixels to be either contour or texture, in line with our required experimental design. Finally, although we are measuring metabolic signals there is good evidence that contrast is unimportant in either contour (Hess et al., 1998) or texture (Schofield and Yates, 2005) perception. In the former, it is thought that a separate code is used for contrast and contours (Hess et al., 1998) and in the latter it is believed that, due to an intensive non-linearity (Graham and Sutter, 1996, 1998; Graham et al., 1992; Wilson, 1993), the role of contrast in texture processing is weak.

Only three kinds of images were used in the actual fMRI experiments: 1) full binary pseudo-natural images and trinary pseudo-natural images derived using 2) maximal or 3) minimal regions of local orientation energy (Figs. 2E,F,G respectively). We will refer to these image categories as “full”, “contour” and “texture” images, respectively, corresponding to the predominant perceptual quality of the images. The contour and texture images are equated in all their low-level statistics (see Table 1). The full images differ from the other two kinds of images in the amount of contrast-energy (root-mean-square or RMS contrast) and sparseness. Thus, there are 4 low-level image properties or variables to correlate the results with, i.e. amount of contrast-energy, degree of sparseness, amount of contours and textures.

Our images are closely matched for global (second-order) statistics and we also have reason to believe that they are fairly well matched for another statistical property that is likely to contribute to the fMRI response: local contrast. To quantify local contrast in these patterns we adapted a methodology described in Mante et al. (Mante et al., 2005). Specifically, we computed the local luminance standard deviation (or local RMS contrast) within a Gaussian-weighted spatial window. The result of this computation on a typical natural image (Fig. 1A) is shown in Fig. 1B, where high pixel values indicate high local contrast-energy in the scene. We applied this methodology to our pseudo-natural images, using various spatial extents of the Gaussian weighting window. To summarize the results we computed the average local contrast-energy (pooled across the whole image) for each image category. We plot this average local contrast-energy as a function of the spatial extent of the Gaussian weighting windows in Fig. 2H, where we also indicate the typical V1 receptive field size near the average eccentricity within our patterns (about 5 deg) derived using high-contrast stimuli (Sceniak et al., 1999). Besides the average local contrast-energy of the full, textured and contour stimuli, we also include the average local contrast-energy of random-half images (where a random-half of the image is set to mean luminance, leaving random parts of both contour and texture structure visible — see synthetic images for details, Fig. 5). Clearly the full images have higher local contrast-energy across all scales of analysis. Note that the contour images have higher local contrast-energy than the texture images and that the random-half images are intermediate, but that this difference is small compared to the difference between half and full images. The origin of small differences in RMS contrast that persist through to large region sizes is due to the higher probability that light or dark pixels will be over-represented in textured regions than in contoured regions (e.g. examine the regions of sky in Fig. 2G) which in turn drives down the local standard deviation. This difference likely arises from the presence of near-uniform luminance surfaces in our images that will be classified as “texture”. We provide a control condition for this later, using “random-half” synthetic images.

The different stimulus conditions were alternated in a block design (block duration = 18 s). Each condition (block) was repeated at least 4 times giving a total duration of ~ 6 min per scan. The stimuli were presented time-locked to the acquisition of fMRI time frames, i.e. every 3 s. To control for attention, the subjects continuously performed a two-interval forced-choice (2IFC) contrast-discrimination task. That is, a given stimulus presentation consisted of two intervals, both displaying a different image from the same condition either at 100% or 60% Michelson contrast. The subject was required to indicate which interval contained the high-contrast stimulus. Each image was presented for 500 ms and the inter-stimulus interval was 250 ms. In the remaining 1.75 s the subjects' responses were recorded. During mean luminance (blank) conditions an identical task was performed for the fixation dot. Virtually all subjects' responses ranged between 90% and 100% correct (See Table 2). Standard retinotopic stimuli were used to create polar-angle and eccentricity maps of the visual cortex (Dumoulin et al., 2003; Engel et al., 1994; Sereno et al., 1995; Wandell et al., 2007).

### Magnetic resonance imaging

The magnetic resonance images were acquired with a Siemens Sonata 1.5 T MRI. The experiments were conducted with the subject lying on their back with a surface-coil (circularly polarized, receive only) centered over their occipital pole. Head position was fixed by means of a foam head-rest and a bite-bar.

Multislice T2⁎-weighted gradient echo (GE) echo-planar imaging (EPI) functional MR images (TR/TE = 3000/51 ms, flip angle = 90°, #slices = 25 (contiguous), slice thickness = 4 mm) were acquired using a surface-coil (receive only) with a 64 × 64 acquisition matrix and a 256 × 256 mm rectangular field of view. The slices were taken parallel to the calcarine sulcus and covered the entire occipital and parietal lobes and large dorsal–posterior parts of the temporal and frontal lobes. One hundred and twenty eight measurements (time frames) were acquired. Eight to twelve fMRI scans were performed in each session. T1-weighted anatomical MR images (aMRI) were acquired prior to the commencement of the functional scans. This aMRI utilized a 3-D GE sequence (TR = 22 ms, TE = 9.2 ms, flip angle = 30°, 256 × 256 mm rFOV) and yielded 80 sagittal images with a thickness of 2 mm.

In separate sessions T1-weighted aMRI images were acquired with a head-coil, also with a 3-D GE sequence, yielding 160 sagittal images comprising 1 mm voxels. Identification of the visual field maps was also performed in another separate session with identical parameters. All studies were performed with the informed consent of the subjects and were approved by the Montréal Neurological Institute Research Ethics Committee.

### Processing of anatomical images

The anatomical MRI scans were corrected for intensity non-uniformity (Sled et al., 1998) and automatically registered (Collins et al., 1994) in a stereotaxic space (Talairach and Tournoux, 1988). The surface-coil aMRI, taken with the functional images, was aligned with the head-coil aMRI, thereby allowing an alignment of the functional data with a head-coil MRI and subsequently stereotaxic space. This alignment was performed with an automated script combining correction for the intensity gradient in the surface-coil aMRI (Sled et al., 1998) and intra-subject registration (Collins et al., 1994). A validation of this method was described in a previous study (Dumoulin et al., 2000). The aMRIs were classified into gray-matter, white-matter and CSF (Cocosco et al., 2003), after which two cortical surfaces were automatically reconstructed at the inner and outer edge of the cortex (MacDonald et al., 2000). The surface-normals of the cortical models were smoothed to produce an “unfolded” model of the cortical sheet (MacDonald et al., 2000). All processing steps were completely automatic and all the data are presented in a stereotaxic space (Collins et al., 1994; Talairach and Tournoux, 1988).

### Preprocessing of functional images

The first 2 time frames of each functional run were discarded due to start-up magnetization transients in the data. All remaining time frames were blurred with an isotropic 3D Gaussian kernel (full-width-half-maximum = 6 mm) to attenuate high frequency noise. The functional scans were corrected for subject motion within and between fMRI scans (Collins et al., 1994). Functional scans were excluded from further analysis if artifacts were found (e.g. large subject motion or spurious spikes). In total 3 out of 87 fMRI scans were excluded from further analysis, primarily due to large subject motion.

### Identification of visual field maps

Early visual cortical visual field maps were identified for every subject using volumetric phase-encoded retinotopic mapping (Dumoulin et al., 2003). The visual field signs of different visual field maps could be identified by combining eccentricity and polar-angle phase-maps (Engel et al., 1994) with the anatomical MRI. Neighboring visual field maps could be identified due to opposite field signs; i.e. V1, V2, V3/VP, V3a and hV4 (Dumoulin et al., 2003; Sereno et al., 1995; Wandell et al., 2007). We use the term hV4 to acknowledge that the macaque human homology of this region is disputed (Brewer et al., 2005; Wandell et al., 2007). In addition to these retinotopic defined visual field maps, we defined a region in the ventral occipital cortex (VO-). This region was defined for every subject as a region in ventral occipital cortex that responded to all stimuli conditions (*t* > 3) outside of the identified visual field maps (indicated with the minus). Thus VO- was not delineated based on retinotopic criteria but defined as a stimulus-responsive region in the ventral occipital cortex and likely contains several visual field maps, e.g. VO-1 and VO-2 (Brewer et al., 2005; Wandell et al., 2007).

### Statistical analysis

The fMRI data were analyzed using software developed by Worsley et al. (Worsley et al., 2002). This statistical analysis is based on a linear model with correlated errors. Runs, sessions and subjects were combined using a linear model with fixed effects and standard deviations taken from the previous analysis on individual runs. A random effects analysis was performed by first estimating the ratio of the random effects variance to the fixed effects variance, then regularizing this ratio by with a Gaussian filter. The variance of the effect was then estimated by the smoothed ratio multiplied by the fixed effects variance to achieve higher degrees of freedom. The resulting *t*-statistical images were thresholded for peaks and cluster sizes using random field theory (Worsley et al., 1996).

**Fig. 3:**
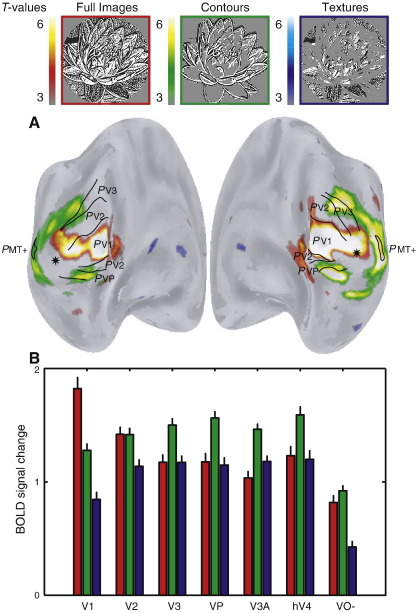
(A) Average *t*-statistical maps (5 subjects) displayed on their unfolded average cortical surfaces (*t* = 5.2 corresponds to *p* = 0.05 corrected for multiple comparisons). FMRI activation elicited by one image category is compared to both others. Oblique posterior-medial views are shown of the left and right hemispheres. These views reveal all differential activations. Black lines indicate the average borders of the visual field maps, thereby describing their probabilistic (*P*) location (Dumoulin et al., 2000, 2003). A star indicates the foveal representation. As can be seen from these t-statistical maps, early visual cortex (V1) respond strongest to full images, whereas later visual cortex (V3 and up) are activated more by the contour-only images. (B) Average BOLD signal amplitudes and standard deviations elicited by different image categories are plotted for the different visual areas. The visual field maps (V1–hV4) were identified in each subject. hMT+ was not identified in every subject and is therefore not included in the VOI analysis. The ventro-lateral occipital–temporal activations that were not covered by the identified visual field maps are grouped as region VO-. These results reveal two different response patterns that dissociate V1 from the rest of the visual cortex. V1 responds strongest to the full images, whereas later visual cortex is activated stronger by the contour images.

The volume of interest analysis (VOI) of the identified visual areas (V1 to VO) was done in an identical fashion (Worsley et al., 2002). These visual areas were identified in each subject. Prior to the VOI analysis, the time-series were converted to percent BOLD signal change and all the time-series of voxels responding to all stimuli within a VOI (left and right hemispheres) were averaged together, with exclusion of voxels displaying artifacts. Because the time-series were converted to percent BOLD signal change prior to the analysis, the effect size of the linear model (β) is also in percent signal change. The effects sizes and their standard deviations, averaged across all subjects, of each condition relative to the overall mean of the time-series are plotted in Figs. 3 and 5.

## Results

### Pseudo-natural images

The functional MRI results are illustrated on unfolded cortical surfaces in Fig. 3A. The *t*-statistical scores indicate regions responding more strongly to a particular image category compared to both other conditions. The data in Fig. 3A is averaged in a stereotaxic space for all subjects (Collins et al., 1994; Talairach and Tournoux, 1988). The data indicate that early visual cortex (approximately limited to V1) respond strongest to the viewing of full images, whereas the fMRI signal is driven more by the contour images in extra-striate visual cortex. No cortical region is activated selectively by the texture images.

A volume of interest (VOI) analysis (Fig. 3B) on the individually identified visual field maps (Dumoulin et al., 2003) confirms the observations of the *t*-statistical maps. The VOI analysis respects the variability of location and size of these areas across subjects. The VOI analysis indicates that the response profile of V1 closely corresponds to the amount of contrast-energy that is present in the stimulus (V1: *t* = 7.7, *p* < 0.001, see Table 1). However in extra-striate visual cortex the strongest response is elicited by the contour-only images (V3–hV4: *t* > 4.4, *p* < 0.001). This is true irrespective of the amount of contrast-energy in the images since the response to the full images and texture images, which strongly differ in their contrast-energy, is roughly equal. Extra-striate cortex respond strongest to images with a large degree of sparseness and contours. Neither attribute alone seems to be responsible since the full images contain the same contours and the texture images have the same degree of sparseness.

### Synthetic images

**Fig. 4:**
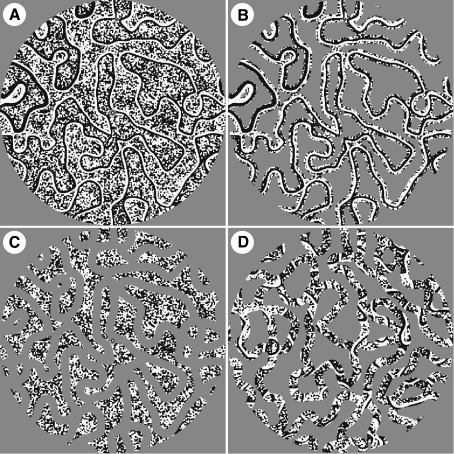
Examples of synthetic visual stimuli used in the control experiment. (A) A binary “Full Image” containing both contours and textures, contour-only and texture-only images are shown in panels B and C, respectively. Panel (D) illustrates an image where a random-half is masked out. Thus this image contains both contours and texture but a sparseness and contrast-energy similar to the contour-only and texture-only images. These images have the same statistical properties as the pseudo-natural images (Fig. 2), but have no recognizable content.

To assess the extent to which our results might depend on the presence of recognizable structure within our “pseudo-natural” images, we repeated the experiment with synthetic images (*n* = 200, Fig. 4). The synthetic images have closely matched low-level statistical properties as the pseudo-natural image categories but without recognizable content. Thus the main difference between the image-sets is that the pseudo-natural images contain recognizable content whereas the synthetic images do not (see Supplementary Table 1 for a summary of several statistical properties).

**Fig. 5:**
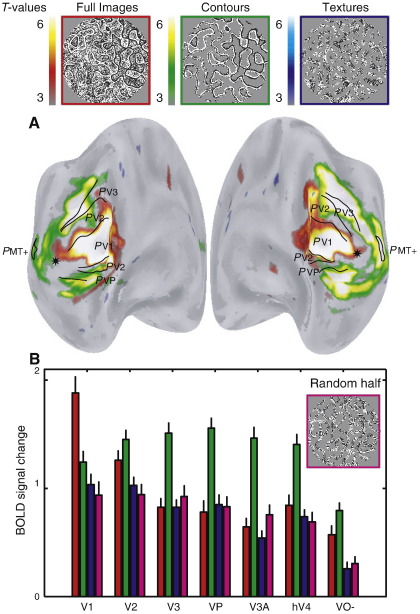
(A) Similar to Fig. 3A, but now for the statistical maps derived from stimulation by the synthetic images (3 subjects). These results are very similar to those from pseudo-natural image viewing, indicating that this result does not depend on recognizable image content. (B) Similar to Fig. 3B, but now for the fMRI signals elicited by the synthetic images. These results with synthetic images replicate the earlier finding with pseudo-natural images that V1 responds strongest to the full images and later visual cortex is driven more by the contour images. This suggests that the results are not dependent on recognizable image content. Furthermore, an additional control image category (random-half images) suggests that the response profiles are not explained by slight differences in the power spectra of the images (see Fig. 6).

As can be seen from both the average *t*-statistical maps (Fig. 5A) and the corresponding VOI analysis (Fig. 5B), the fMRI signal profile driven by the synthetic images is similar and even more pronounced than that elicited by the pseudo-natural images. Again, V1 responds strongest to full images (VOI analysis: *t* = 5.7, *p* < 0.001) and strongest extra-striate responses are elicited by contour images (VOI analysis V3–hV4: *t* > 6.2, *p* < 0.001). That the effect is even clearer in synthetic images is likely attributable to the more pronounced dissociation of contours- and texture-structure in the synthetic versus pseudo-natural images. The similarity of the pattern of results obtained in the pseudo-natural and synthetic image conditions suggests that the results are independent of recognizable image content.

**Fig. 6:**
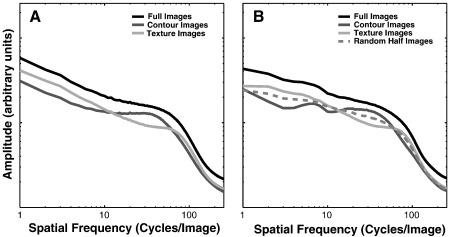
Average power spectra (*n* = 200) of the pseudo-natural (A) and synthetic (B) image categories.

The binarization process led to several statistical properties of the images containing mainly contours or texture being near identical (see Table 1). The power spectra of these two conditions are also very similar (see Fig. 6) as are their average local contrast (see Fig. 2H). However, we wondered whether a subtle difference in the power spectra, local contrast or perhaps any other yet unconsidered statistical property, might explain the difference in fMRI signal obtained with contour and texture images. To assess this, images were created where a random-half was set to mean luminance (Fig. 4D, referred to as “random-half” images). The power spectra, and other statistical properties we measured, of these random-half images are in between that of the contour and texture images (see Fig. 2H, Fig. 6B, and Supplementary Table 1). Therefore, if the results were due to the differences in power spectra, local contrast or any other statistical property, then, assuming a linear system, the fMRI results driven by the random-half images should be intermediate as well. The fMRI signal amplitude to the random-half images is similar to the texture images (Fig. 5B). Therefore, the pattern of results are most likely due to neither the slight differences in their power spectra, nor variations in local RMS contrast (nor any other related linear image statistic). Furthermore, it suggests that the presence or absence of contours is mediating the responses to contour and texture images.

### Correlation between fMRI and image statistics

**Supplementary figure 1:**
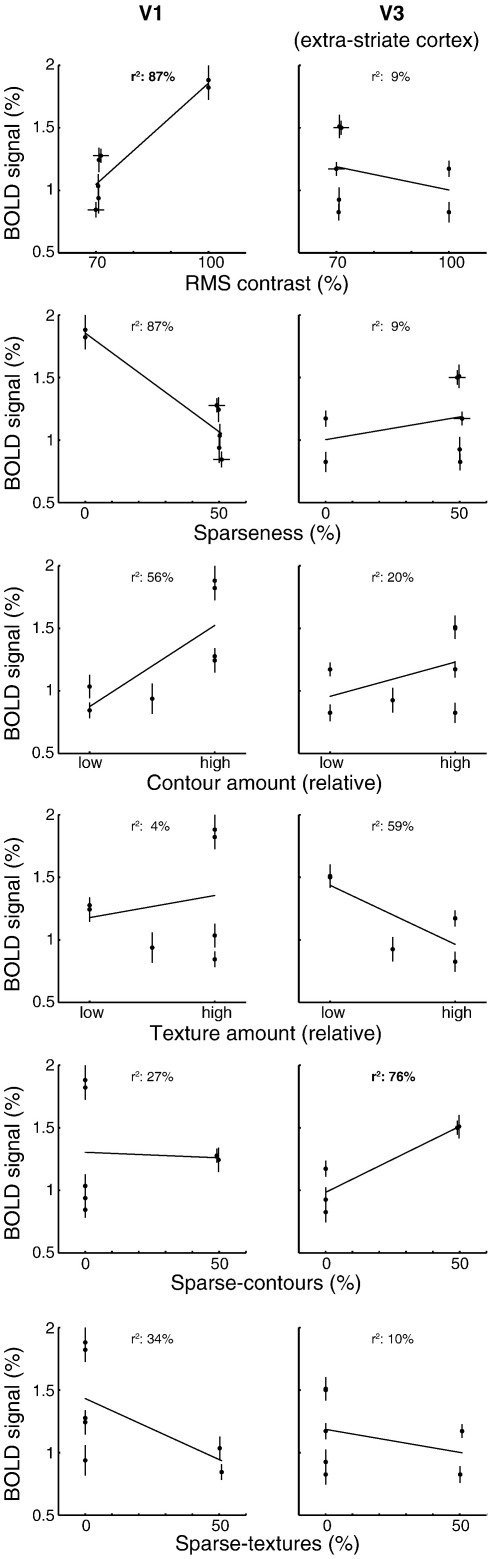
All correlations between fMRI BOLD signal amplitudes and image properties (see Fig. 7).

**Fig. 7:**
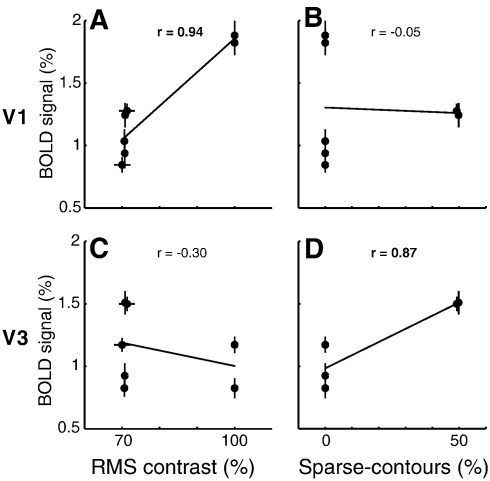
Correlations between fMRI BOLD signal amplitudes elicited by our stimuli and their image properties. The correlations are shown for visual field maps V1 (A, B) and V3 (C, D). We only show the image properties that explain most of the variance in both visual field maps, i.e. contrast-energy (A, C) and the presence of sparse-contours (B, D). For more extensive comparisons with other image properties see Supplementary Figure 1. Each correlation is based on 7 different image types and the matching fMRI BOLD response amplitudes (Figs. 3 and 5). The correlation analysis shows that 87% of the V1 amplitude variance is explained by contrast-energy, whereas 78% of the V3 variance is explained by the presence or absence of sparse-contours.

We directly correlated the fMRI signal amplitudes with the different image properties, contrast-energy, sparseness, and the amount of contours and textures (Fig. 7). Measurements of the first two image properties were taken from Table 1 and Supplementary Table 1, whereas, the latter two were determined by the image construction process (binary values were used except for the random-half image category: 0.5). We also incorporated interactions between these image properties, i.e. sparse-contours and sparse-textures. These interactions were calculated by multiplying sparseness measurements by a binary indicator of the presence of extended contour/texture information (random-half images were treated as having neither extended contour nor texture information). For all comparisons see Supplementary Figure 1.

In V1, the 87% of the fMRI amplitude variations (*r*^2^) is explained by the contrast-energy in the images (Fig. 7A). In our images contrast-energy and sparseness are inversely correlated, therefore sparseness also captures 87% of the variance in the data (*r* = − 0.94). Percent variance explained by the amount of contours (56%) and textures (4%) is less (see Supplementary Figure 1 for a graphic of all comparisons). Using a stepwise regression, only contrast-energy (87%, *p* << 0.001) and contour amount (9%, *p* = 0.04) or, if included, sparse-contours (11%, *p* = 0.01), contributed significantly to explain the total variance (96% or 98%) in the data. The percent variance explained can differ for stepwise regression compared to separate regression of each image property, because in separate regression the total variance is minimized by one image property whereas in stepwise regression the image properties are sequentially combined to minimize the total variance.

The response profiles in extra-striate cortex (V3–hV4) were similar (Figs. 3B and 5B), therefore we compared the signal amplitudes of one early representative visual field map (V3) with the different image properties (Figs. 7C and D, and see Supplementary Figure 1). Unlike V1, the percent variance explained by contrast-energy is small (9%, Fig. 7C) and inversely correlated (*r* = − 0.30). This analysis assumes a linear relationship, but we suspect that a biologically plausible non-linear relationship would also fail to capture the large range of fMRI signal amplitudes within little or no contrast variations. An interaction between sparseness and the presence of contours explains most of the variance in the data in extra-striate cortex (78%, Fig. 7D) but not V1 (0.3% or 11% in separate or stepwise regression, Fig. 7B). Percent variance explained by sparseness (9%) and the amount of contours (20%) and textures (59%) is less. Using a stepwise regression and including our interactions, only sparse-contours (76%, *p* = 0.01) contributed significantly to explain the variance in the data.

### Detection thresholds of the images

**Fig. 8:**
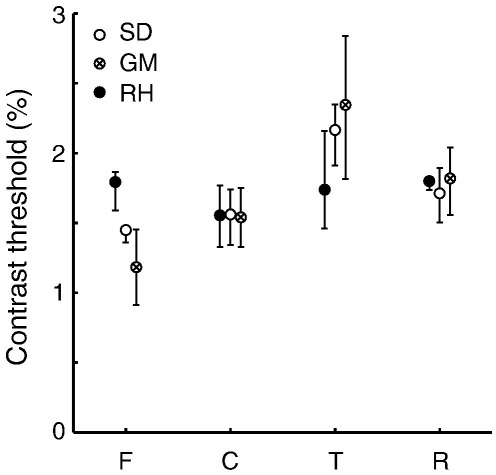
Average psychophysical detection thresholds and 95% confidence intervals of three subjects for the synthetic image categories: full (F), contour (C), texture (T) and random-half (R) images. The subjects' average thresholds (1.4, 1.8, and 1.9% respectively) were aligned to the overall mean (1.7%) for visualization purposes. For two subjects (SD, GM), the detection thresholds of texture images are higher than for the other image categories. The detection thresholds are similar for the full, contour and random-half images.

To assess whether the activation profile in striate and extra-striate cortex correlated with perception, we estimated psychophysical detection thresholds for the synthetic images (Fig. 8). The images were presented using Psykinematix (KyberVision, Montréal, Canada) on a Macintosh G4 Powerbook, and displayed on a CRT monitor (Mitsubishi Diamond Pro 2070SB). The viewing conditions were identical as in the fMRI experiments, with two exceptions: 1) the presentation duration (0.1 s) and 2) we masked the central 3 deg of the stimulus, because in the central representation was not included in our fMRI VOI analysis due to conventional phase-encoded retinotopic mapping limitations (Dumoulin and Wandell, 2008; Wandell et al., 2007).

The psychophysical procedure consisted of two presentations; one contained a stimulus the other only mean luminance. The subject indicated which presentation contained the stimulus (1 or 2). The stimuli were presented at 9 roughly equally spaced levels between 0 and 3.9% Michelson contrast. The subjects' performance was fitted with a Weibull function and we determined the contrast detection threshold at 75% correct (Wichmann and Hill, 2001a). We computed the 95% confidence intervals of these thresholds using a bootstrap procedure (Wichmann and Hill, 2001b). Three subjects participated in the psychophysical procedure including one (GM) who was naive to the purpose of this study.

The results for three subjects and all stimulus types are similar (Fig. 8). In two subjects, significantly more contrast is required to detect the texture images compared to the other stimuli. The thresholds for full, contour and random-half images were similar.

The V1 and extra-striate response profile differences are primarily mediated by a change in the response to full and contour images. These response differences were not reflected in the psychophysical detection thresholds. Perception does not need to correlate with signals in specific visual areas. Furthermore, these psychophysical estimates at detection threshold may not reflect supra-threshold behavior. Lastly, different psychophysical or behavioral procedures may yield measures that correlate with the fMRI signal amplitudes.

## Discussion

### Summary

The results reveal a distinct response pattern to complex images in striate and extra-striate visual cortex. V1 responds the strongest to full images, whereas the strongest fMRI responses in extra-striate visual cortex are elicited by sparse images containing contours. These results in extra-striate cortex are independent of contrast-energy (Figs. 3, 5 and 7), recognizable image content (Fig. 5), slight differences in power spectra (Figs. 5 and 6) or psychophysical detection thresholds (Fig. 8). Thus, in answer to the question posed in the introduction, image sparseness does influence the response of later visual cortex but only when associated with contour structure.

### V1

Most of the variance in the V1 responses to our complex images is explained by the amount of contrast-energy in the images. This is in agreement with the general notion that V1 represents the outside world using the response of many local filters (De Valois and De Valois, 1990), as well as more specific proposals that the V1 population response, as measured with optical imaging (Basole et al., 2003), accords with the spatio-temporal contrast-energy of the stimulus (Mante and Carandini, 2005). Furthermore, it is broadly consistent with the finding that fMRI signals in early visual cortex correspond with the contrast (energy) of spatially narrowband stimuli (Boynton et al., 1999) as well as broadband natural images (Olman et al., 2004).

Even though V1 responses are broadly consistent with the contrast-energy within the images, we observed a significant contribution of contour information to the V1 response. This contribution is larger for pseudo-natural than for synthetic images (see Figs. 3 and 5). This difference may be attributed to a difference in contour-statistics, such as edge co-occurrence (Geisler et al., 2001). Alternatively, this V1 difference may be attributed to the distribution of the texture “patches”. In the synthetic images they are less extended than in the pseudo-natural images, this may yield a different fMRI response because, while they occupy 50% of the image, they are more sparsely distributed. We speculate that the modest elevation of activity in V1 associated with contour information reflects facilitative interactions tuned for local contour structure (Gilbert et al., 1996), and/or suppressive center-surround interactions for broadly distributed local orientation structure (Allman et al., 1985; Fitzpatrick, 2000).

### Extra-striate visual cortex

Extra-striate visual cortex responded the strongest to images containing sparsely distributed contours, independent of amount of contrast-energy or recognizable image content. This preference in extra-striate cortex extends into cortical regions associated with object processing (Grill-Spector et al., 2001; Malach et al., 1995) and, perhaps more surprisingly, into regions not traditionally associated with object processing (e.g. hMT+) (Dumoulin et al., 2000; Tootell et al., 1995; Watson et al., 1993). We speculate that this preference occurs relatively early in the visual processing sequence and is then imposed on later visual cortex.

We use complex images derived from natural scenes. These complex images vary across many dimensions that could be responsible for the strong response to the images containing sparsely distributed contours. We have excluded several properties such as contrast-energy, recognizable image content, power spectra and psychophysical detection thresholds. Therefore, we believe that the most parsimonious explanation is that the presence of sparse-contours dominates the responses to complex scenes in extra-striate cortex.

The strong response to contour images compared to the full images is counter-intuitive. First, the two image categories are similar in their detection threshold. Second, the two image categories have identical contours. Similar psychophysical thresholds as well as identical contours suggest a similar perceived shape and some of these extra-striate regions have been proposed to encode perceived shape rather than image features (Kourtzi and Kanwisher, 2001). Lastly, removing contrast-energy from the images can actually boost the fMRI signal in extra-striate cortex.

If later visual cortex responds selectively to contour structure (or perceived shape) why should removing contrast-energy from the images increase the fMRI signal amplitude in extra-striate cortex? We propose that this boost may be mediated by both suppressive and facilitative interactions. We will discuss both interactions sequentially starting with possible suppressive interactions.

The response to the full images is decreased in extra-striate cortex (see Figs. 3 and 5), thus the presence of textures within the scene seem to decrease the cortical responses. This phenomenon could be attributed to established neuronal processes, such as contrast normalization (Carandini et al., 1997; Heeger, 1992) or surround suppression (Allman et al., 1985; Fitzpatrick, 2000) operating between V1 and V3/VP. Surround suppression – a reduction in response arising from the introduction of a surround to a visual target – has been found to decrease fMRI signals when using narrowband stimuli (Williams et al., 2003; Zenger-Landolt and Heeger, 2003) and broadband stimuli patches (Kastner et al., 2001) in all visual areas (for other extra-classical receptive field effects using fMRI see also Dumoulin and Hess, 2006; Harrison et al., 2007; Murray et al., 2002). Some of these previous fMRI studies (Kastner et al., 2001; Zenger-Landolt and Heeger, 2003) describe stronger surround suppression in extra-striate than striate cortex. In these studies, the surround was spatially segregated from the target region, to a degree that the changes in fMRI activation of a particular target can be measured separately from those directly elicited by the surround. This is not the case in our study. We did not have the spatial resolution to specifically measure the effect of the surround regions (textures) on target regions (contours) when the two were presented simultaneously (full images). Thus, the lower response to full images in extra-striate cortex can be interpreted as strong surround suppression that may originate from the close spatial proximity of the different image features and the broadband nature of our images, where orientation, spatial phase and spatial frequency effects are all intermixed.

Besides the decrease in fMRI signal for full images in extra-striate cortex, we observe an increase in the fMRI response for contours images, especially when compared to texture images (or random-half images). Extra-striate response is equally weak to isolated textures, random-half images and full images. Thus, besides suppressive mechanisms, we see evidence for specific amplification of sparse-contour structure. Processes such as perceptual grouping (Grossberg et al., 1997), flank facilitation (Polat and Sagi, 1993) or contour integration (Field et al., 1993) may mediate this amplification.

**Supplementary figure 2:**
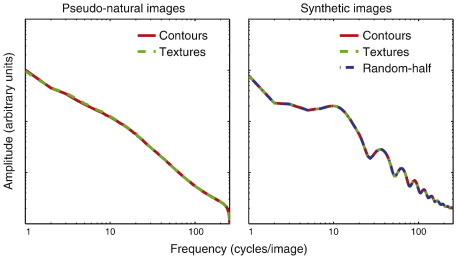
Average contrast-modulated power spectra (*n* = 200) of the pseudo-natural and synthetic image categories. Contrast-modulated structure was estimated by taking the absolute value relative to mean luminance. The full images are not included because according to our computation they do not contain any contrast-modulated structure. There are no differences in contrast-modulated power spectra between any of the stimulus conditions.

Alternatively, sparseness in the half-images introduces contrast-modulated (“second-order”) structure, which later visual cortex is proposed to process more (e.g. Baker, 1999). This contrast-modulated structure is similar for the different half-images (see Supplementary Figure 2) and on its own cannot explain an enhancement of the response to one of the half-images (contour images). However, contrast-modulated structure may boost the contour structure when they are aligned. Alignment of contrast-modulated and contour (luminance) information frequently occurs in natural images (Johnson and Baker, 2004) and has been proposed to increase perceptual accuracy (Smith and Scott-Samuel, 2001).

Thus, we propose that both suppressive and facilitative interactions underlie the fMRI signals in extra-striate cortex. Several known mechanisms may mediate these suppressive and facilitative interactions, with the current results we cannot discriminate between these specific mechanisms. It is clear however, that these mechanisms operate not on one specific image property but on a combination related to image sparseness and contour information.

### Phase structure

Because our images were equated for simple luminance statistics the co-dependencies in luminance that lead to contours or texture are captured entirely by the phase structure of the image. Thus, our finding of lower responses to textures than contours can be linked to Rainer et al.'s (Rainer et al., 2001) finding that phase-scrambled natural scenes (which are textures) elicit lower BOLD MRI response in V1, STS and inferior temporal cortex of anesthetized macaque, than the original images (which are perceptually dominated by contour structure). However this finding runs contrary to Olman et al. (Olman et al., 2004) who report that phase-scrambling stimuli has no effect on the response of primary visual cortex to natural scenes. We speculate that the most likely cause of this discrepancy is Olman et al.'s use of images from the Van Hateren database (van Hateren and van der Schaaf, 1998). Many of these images are heavily textured forest scenes, containing few objects apart from trees, from which Olman et al. then selected those that had “predominantly unimodal pixel intensity histogram distributions” which may also favor the selection of textures. By contrast in both this study and that of Rainer et al., images were selected that were rich in objects (both natural and man-made) and therefore much more rich in contours.

### Attention and eye-movements

Attentional modulation can substantially affect neuroimaging responses in visual cortex (Beauchamp et al., 1997; Brefczynski and DeYoe, 1999; Gandhi et al., 1999; Martinez et al., 1999; Murray and Wojciulik, 2004; O'Craven et al., 1997), and could potentially confound the interpretation of the results. We do not believe that different levels of attention to the different image categories can explain our results for the following reasons. Firstly, we controlled for attention by our contrast-discrimination task. Performance for this task was virtually identical for each image category. Similar task performance supports the notion that the level of attention was similar for the different image categories; furthermore this task directed attention towards the images yielding increasing the specificity of the neural response (Murray and Wojciulik, 2004).

Secondly, the results are inconsistent with current observations about the effect of attention on the fMRI signals. Current evidence indicates a gain increase for the attended stimuli (Beauchamp et al., 1997; Brefczynski and DeYoe, 1999; Gandhi et al., 1999; Martinez et al., 1999; Murray and Wojciulik, 2004; O'Craven et al., 1997; Somers et al., 1999). We do not find evidence for such an attention-mediated signal increase. Specifically, in our experiments increased attention towards a particular image category would boost the fMRI response to that image category. For example, increased attention towards the contour images would boost the response to the contour images. This attention-based explanation could account for the responses in extra-striate cortex, but not V1. Yet attention is known to modulate V1 responses as well (Brefczynski and DeYoe, 1999; Gandhi et al., 1999; Martinez et al., 1999; Somers et al., 1999). A similar argument based upon increased attention towards the full images may explain the V1 signals but will fail in extra-striate cortex. Thus, differential activation patterns in V1 versus extra-striate cortex make any explanation based on attention unlikely.

In our experiment we did not monitor eye-movements but we think it unlikely that eye-movements played a significant role in determining our results for the following reasons. Firstly, the subjects were experienced psychophysical observers instructed to fixate on the provided fixation point. Secondly, we have demonstrated that (largely the same) subjects are able to maintain fixation during an identical task with different structured and unstructured images (Dumoulin and Hess, 2006). Thirdly, the regions of the cortical network associated with eye-movements (Krauzlis, 2005; Pierrot-Deseilligny et al., 2004) were not seen in this study. Therefore, we believe that sensory, rather than attention or eye-movement-related, processes are underlying our results.

## Conclusion

In summary, we have argued that our results are consistent with an initial representation of the natural scenes in V1 based on local oriented filters and incorporating modest facilitation of structure that is consistent with the presence of contours. We propose that reduced activation of later visual cortex to full scenes (compared to contour-isolated stimuli) results from suppressive interactions between texture and contour processing mechanisms. We have also argued that selective amplification of response to sparse-contour images in extra-striate cortex is consistent with the operation of contour enhancement mechanisms pooling responses from earlier visual cortex to signal more complex image structures.

## Figures and Tables

**Table 1:** Statistical summary of the pseudo-natural stimuli

		“Full images”	“Contours”	“Textures”
Contrast:	Rms	100(0)%	71.3(2.1)%	70.0(2.2)%
Michelson		100(0)%	100(0)%	100(0)%
Sparseness:		0(0)%	49.1(3.1)%	50.9(3.1)%

Luminance histogram:				
	White	50.6(2.2)%	25.0(1.8)%	24.4(2.2)%
	Gray	0(0)%	49.1(3.1)%	50.9(3.1)%
	Black	49.4(2.2)%	25.9(1.9)%	24.7(2.2)%

The table lists several statistical measurements (average and standard deviations) for the different stimulus conditions. The statistical summary of the synthetic images is similar but with even smaller differences between the images (see supplementary Table 1). Image sparseness is simply defined by the percentage of mean-luminance pixels. The table illustrates that the “contour” and “texture” conditions were similar for all low-level statistical measures. Their luminance histograms are nearly identical; therefore all statistics derived from the histogram will be similar.

**Table 2:** Average percent correct and standard deviations of the subjects on the contrast discrimination task

	“Full images”	“Contours”	“Textures”	“Random-half”
Pseudo-natural images	0.97(0.05)	0.98(0.04)	0.96(0.04)	–
Synthetic images	0.96(0.05)	0.97(0.06)	0.97(0.04)	0.98(0.06)

No significant differences between any conditions were found (ANOVA, *p* > 0.1).
