# Supplementary material for: Sparsely distributed contours dominate extra-striate responses to complex scenes
Source: Neuroimage. 2008 Aug 15;42(2):890–901. doi: 10.1016/j.neuroimage.2008.04.266 (PMC2572731; doi:10.1016/j.neuroimage.2008.04.266)
Supplement: Supplementary file 1 [file mmc1.pdf]

		"Full images"	"Contours"	"Textures"	"Random-half"
Contrast:	rms	100(0)%	70.8(0.5)%	70.6(0.5)%	70.7(0.5)%
	Michelson	100(0)%	100(0)%	100(0)%	100(0)%
Sparseness:		0(0)%	49.8(0.7)%	50.2(0.7)%	50.0(0.7)%
Luminance histogram:					
	white	50.1(1.3)%	25.1(1.3)%	25.0(0.5)%	25.0(1.4)%
	gray	0(0)%	49.8(0.7)%	50.2(0.7)%	50.0(0.7)%
	black	49.9(1.3)%	25.1(1.4)%	24.8(0.5)%	25.0(1.4)%

Supplementary Table 1: Statistical summary of the synthetic stimuli, similar to Table 1. The synthetic stimuli contained a "random-half" condition, whose statistical properties are in between that of the "contour" and "texture" conditions.
